# Temperature and Strain Correlation of Bridge Parallel Structure Based on Vibrating Wire Strain Sensor

**DOI:** 10.3390/s20030658

**Published:** 2020-01-24

**Authors:** Lu Peng, Genqiang Jing, Zhu Luo, Xin Yuan, Yixu Wang, Bing Zhang

**Affiliations:** 1 National Center of Metrization for Equipments of Roads and Bridges, Research Institute of Highway Ministry of Transport, Beijing 100088, China; gq.jing@rioh.cn (G.J.); ZhuLuo@ncmerb.com (Z.L.); wyx@ncmerb.com (Y.W.); b.zhang@rioh.cn (B.Z.); 2 School of Electrical and Information Engineering, Wuhan Institute of Technology, Wuhan 430205, China; qcmhssk@163.com

**Keywords:** parallel position, bridge structure, temperature, vibrating wire strain sensor

## Abstract

Deformation is a ubiquitous phenomenon in nature. This process usually refers to the change in shape, size, and position of an object in the time and spatial domain under various loads. Under normal circumstances, during engineering construction, technicians are generally required to monitor the safe operation of structural facilities in the transportation field and the health of bridge, because monitoring in the engineering process plays an important role in construction safety. Considering the reliability risk of sensors after a long-time work period, such as signal drift, accurate measurement of strain gauges is inseparable from the value traceability system of high-precision strain gauges. In this study, two vibrating wire strain gauges with the same working principle were measured using the parallel method at similar positions. First, based on the principle of time series, the experiment used high-frequency dynamic acquisition to measure the thermometer strain of two vibrating wire strain gauges. Second, this experiment analyzed the correlation between strain and temperature measured separately. Under the condition of different prestress, this experiment studied the influencing relationship of temperature corresponding variable. In this experiment, the measurement repetitiveness was analyzed using the meteorology knowledge of single sensor data, focused on researching the influence of temperature and prestress effect on sensors by analyzing differences of their measurement results in a specified situation. Then, the reliability and stability of dynamic vibrating wire strain gauge were verified in the experiment. The final conclusion of the experiment is the actual engineering in the later stage. Onsite online meteorology in the application provides support.

## 1. Introduction

Bridge health monitoring and diagnostic discriminant models have always been a key challenge for the transportation sector worldwide. In a previous study, on a regular and irregular basis, construction workers used different monitoring instruments to test some components of structure, analyzed the data, and finally evaluated the performance of structure. In 2018, Mao by monitoring dynamic characteristics of Sutong cable-stayed bridge (SCB), including acceleration and strain responses, as well as modal frequencies, are investigated through one-year continuous monitoring data under operating conditions by the structural health monitoring system. One-year continuous modal frequencies of SCB are identified using the Hilbert–Huang transform method. Variability analysis of the structural modal frequencies due to environmental temperature and operational traffic is then conducted. Results show that temperature is the most important environmental factor for vertical and torsional modal frequencies. The traffic load is the second critical factor especially for the fundamental vertical frequency of SCB [1]. In 2019, Wang reported that the real-time monitoring data collected from a long-span cable-stayed bridge is utilized to demonstrate the feasibility of the improved BDLM-based method. In particular, the present BDLM-based method allows for probabilistic forecasts, offering substantial information about the target TIS response, such as mean and confidence interval. Results show that the improved BDLM is capable of capturing the relationship between temperature and TIS. Compared to the AR model, multiple linear regression (MLR) model, and BDLM without the AR component, the improved BDLM shows better forecasting performance in modeling and forecasting the TIS of a long-span bridge [2]. Such measurement methods have the drawback of large errors and discontinuities. The ideal health monitoring system should accurately reflect the change in grassroot structure under the influence of factors such as temperature, humidity, and other environmental factors, installation and deployment methods, and the sensor’s own error, and establish an effective health assessment and prediction model. For example, Mao proposed according to one-year continuous monitoring of strain data recorded by the structural health monitoring system of the Sutong cable-stayed bridge, the lifetime fatigue reliability of three welded details of the orthotropic steel deck was investigated, detailed analysis of the separated components of the raw strain data was first conducted, and included the slow-varying trend and the dynamic component. The strain dynamic component was mainly induced by the local vehicle axle loads. Rainflow counting was used to obtain the stress range histograms, which were then used to calculate the equivalent stress range according to the lognormal-fitting method. Finally, a time-dependent fatigue reliability evaluation of the described three welded details was conducted using one-year monitoring strain data. Results showed that the fatigue performance of the two welded details, RTDD and DTD, remained satisfactory after 100 years of operation because the failure probabilities were both lower than 10^−5^. The designed cutout of the diaphragm was applied to the RTD weld at the welded connection between the U rib and diaphragm. This cutout was validated as a means to help achieve better fatigue resistance for the RTD weld [3]. For bridge strain monitoring sensors, most experimental procedures use resistive strain gauges, vibrating wire strain gauges, fiber grating strain gauges, etc. Although the development of fiber grating technology in recent years has led to a new round of equipment upgrades for structural monitoring methods, vibrating wire strain gauges are most widely used in stage bridge health monitoring systems.

In recent years, studies on the heat of vibrating wire sensors at home and abroad have continued unabated, and domestic and foreign experts and scholars have conducted a lot of research to improve the sensor performance. The research on sensors is gradually extended towards high precision, large scale, small volume, and multiple applications. During the improvement in the performance of the sensor, higher requirements are also imposed on the measurement accuracy. To improve accuracy, in 2010, He proposed a low-voltage excitation with the feedback method. By pre-excitations, the vibration frequency of the sensor can be used as the output of the driver. The feedback signal frequency is very close to the real frequency of the sensor, so the wire can reach resonance state quickly. The optimal excitation strategy was verified by the new designed detection circuit. The data perform as short-time excitations with large resonance amplitude, therefore the anti-interference ability got enhanced at lower cost of signal processing circuit, finally increasing the measurement time and improving the accuracy of measured frequency [4]. In 2010, Wen proposed a method for the frequency measurement of vibrating wire sensors with LM3S6965 as the control core. An equal precision measurement was used to effectively improve the measurement accuracy of the system [5]. In 2016, Tian et al. proposed a design method of nonlinear compensation that is provided for the nonlinear relation between the measured force and the frequency of vibrating wire sensor. Data density is increased by interpolation based on the principle of cubic spline interpolation. The F-f curve of vibrating wire sensor is fitted and revised by MATLAB based on the least-square method, more accurately the smooth curve according to engineering practice is obtained and a reasonable conclusion is obtained. Experiments show that the method achieves the accurate fitting to the F-f curve of vibrating wire sensor with the limited data. The F-f function of vibrating wire sensor which is long buried can be rapidly determined by this procedure [6]. In 2017, Chen et al. proposed an adaptive vibration measurement method based on fast Fourier transform, which uses digital Fourier filtering to automatically filter noise interference and then uses Quinn algorithm for high-precision frequency calculation. Based on the STM32 processor platform, the method was used in vibrating a wire sensor frequency measurement system. The test results show that the relative error of system frequency measurement is less than 0.01% in the case of no noise interference; in the case of severe white noise interference (signal to noise ratio is −20 dB), the system’s frequency measurement relative error is less than 0.3%. Compared to other frequency measurement methods, it was found that the method has better noise suppression and frequency measurement accuracy [7]. In terms of static metrology and calibration, the experiment proved that a vibrating wire strain gauge can be widely used in the field of geotechnical engineering, and the accuracy requirement for variable measurement is relatively low. However, in the application of traffic engineering, the measurement accuracy is correspondingly improved, accompanied by the needs of metrology and calibration. Many scholars used a strain sensor calibration device consisting of a calibration frame and digital display to calibrate certain metering characteristics of strain gauge. For example, in 2009, Xu invented a vibrating wire strain sensor calibration device that uses a dial gauge as a gauge for deformation, with a deformation range of up to 3 mm and a strain measurement resolution of about 0.1%. The results show that the strain in the middle of the sensitive grid of strain gauge is the largest and gradually decreases to zero at both ends when measuring. The force has no effect on the sensitive gate base layer and bonding layer at both ends and there is a strain transition zone between the base layer and the sensitive gate, the bonding layer, and the base layer. The longer the horizontal width of bonding layer is the thinner the thickness and the larger the shear modulus will be, and the strain transfer will be more efficient if the active zone of the sensitive grid is longer [8]. In 2011, Zhang et al. designed a vibrating wire strain sensor calibration device. They used a grating scale as the measuring standard for deformation. The maximum calibration distance was 300 mm, and the measurement resolution was 2 µm. The degree is about 0.2% [9]. In 2016, Mai et al. invented a vibrating wire strain gauge calibration device. A dial gauge was used as the measurement standard for deformation amount, so that the resolution was 0.01 mm [10]. During engineering application, many in-depth studies have been conducted on the dynamic calibration of strain gauges. In 2016, Bai et al. affixed FBG sensors on standard beams. Loads were applied to the standard beams to deform them, and the beams were measured. The surface strain was used to calibrate the sensitivity coefficient of FBG strain gauge, and the measured strain range could be analyzed up to 2000 µƐ [11]. In 2017, Zhang proposed a method for the indirect calibration of fiber grating strain sensors using a fiber grating temperature sensor. The lossless calibration of strain sensors was achieved [12]. During the detection, monitoring, and metering of vibrating wire strain gauges, the temperature deformation caused by temperature changes has been the research direction of many scientists. Chen et al. monitored data in engineering applications based on the working principle of vibrating wire strain gauges. The relationship between temperature and strain was analyzed using the relevant data of bridge strain monitoring, and the relationship between temperature influence and strain of the string itself and the temperature field of structural section was evaluated [13]. Bai et al. analyzed that when the temperature changes significantly, a variety of mathematical models were used to fit and calibrate the monitoring data, eliminating the temperature drift of strain gauge, and the experimental results reflect the original characteristics of deformation [14]. Agostiono investigation of the sensing features of the long-period fiber gratings (LPGs) fabricated in hollow core photonic crystal fibers (HC-PCFs) by the pressure assisted electric arc discharge (EAD) technique. In particular, the characterization of the LPG in terms of shift in resonant wavelengths and changes in attenuation band depth to the environmental parameters: Strain, temperature, curvature, refractive index, and pressure is presented. Results show that LPGs in HC-PCFs represent a novel high performance sensing platform for measurements of different physical parameters including strain, temperature and, especially, for measurements of environmental pressure. The pressure sensitivity enhancement is about four times greater if comparing LPGs in HC and standard fibers. Moreover, differently from LPGs in standard fibers, these LPGs realized in innovative fibers, i.e., the HC-PCFs, are not sensitive to the surrounding refractive index. During the online calibration study of strain sensors [15], offline removal and reinstallation of sensing elements pose a risk to the continuity and consistency of monitoring data. At present, the load test method is mainly used, but the load test method cannot eliminate the instability of structure such as the stiffness and strength of the structure itself [16,17]. Therefore, a more effective method at this stage is to install a traceable high-frequency dynamic monitoring sensor in parallel to the sensor to perform parallel measurement, thus achieving the online calibration of a long-term monitoring sensor. At present stage, the measurement calibration of the strain sensor system in various industries only adopts static calibration before installation or carries out a static and dynamic load test for calibration, and does not carry out online calibration during use. The static calibration cannot determine the influence of the error and noise, temperature change, and prestress effect on the strain value. Static and dynamic load tests cost a lot of manpower and materials, and cannot provide reference for sensor calibration under complicated passive excitation. In the online calibration research of strain sensors, because the offline disassembly and reinstallation of the sensing elements pose a risk to the continuity and consistency of the monitoring data, the load test method is mainly used at this stage, but the load test cannot eliminate the rigidity and strength of the structure itself.

**Figure 1:**
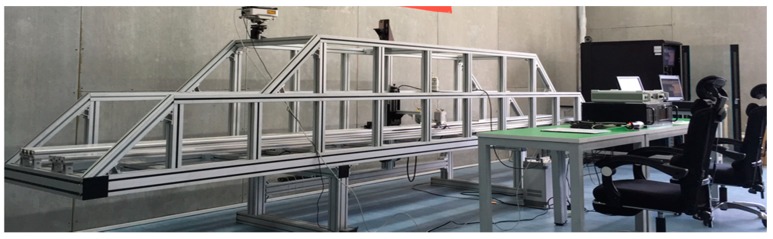
Schematic diagram of simply supported beam test model.

**Figure 2:**
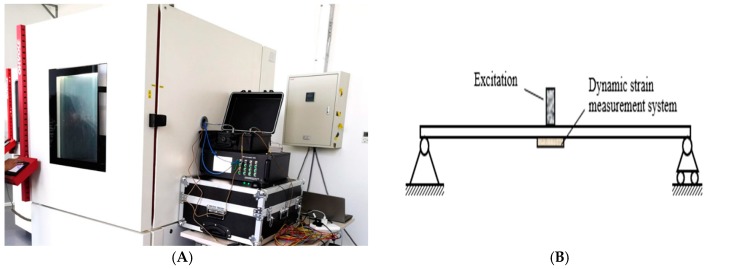
High and low temperature dynamic strain test. (**A**)Test equipment, (**B**) Assembly drawing of experiment beam.

**Figure 3:**
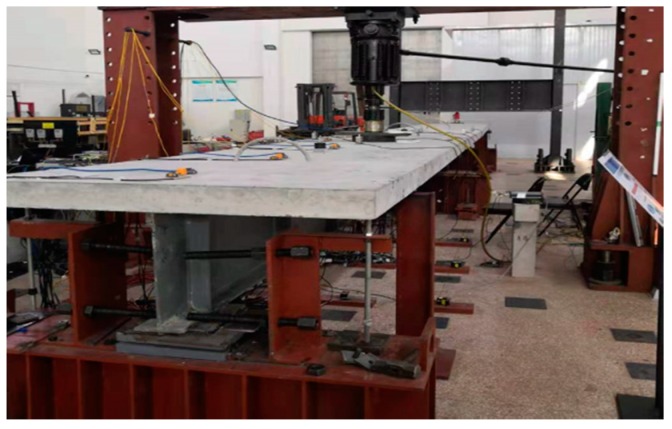
Verified by fatigue tests on bridge model.

**Figure 4:**
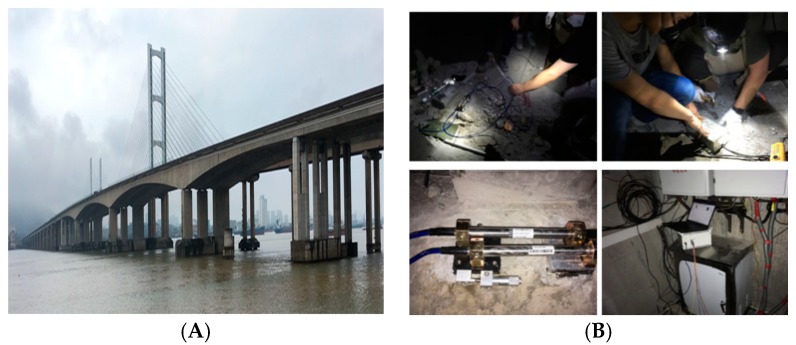
Test verification of Jiujiang Bridge. (**A**) Jiujiang Bridge, (**B**) Test process.

In the research process, we carried out a number of relevant tests. For example, the calibration of fixed excitation by a simply supported beam model is shown in Figure 1. The strain correlation test of a small simply supported beam at constant temperature in a high and low temperature box is shown in Figure 2. Fatigue test verification is shown in Figure 3. In order to further verify the test, a test was carried out on Jiujiang Bridge to verify Figure 4. Most of the above studies adopted common source excitation schemes, mainly to verify the calibration of the strain monitoring system under common source excitation by parallel measurement methods, which provided important verification support for the coupling relationship between temperature, prestress, and strain output in the online calibration of dynamic strain measurement in this study.

During this study, two vibrating wire strain gauges with the same working principle were measured using the parallel method at similar positions. The feasibility of a parallel measurement scheme was verified [18]. Under the condition of different prestresses, this experiment studied the influencing relationship of temperature corresponding variable [19,20]. We focused on researching the influence of temperature and prestress effect on sensors by analyzing differences of their measurement results in a specified situation. The measurement repetitiveness was analyzed using the meteorology knowledge of single sensor data, and then the reliability and stability of a dynamic vibrating wire strain gauge were verified in the experiment. The relevant measurement performance of a dynamic vibrating wire strain gauge was demonstrated.

## 2. Working Principle of Vibrating Wire Strain Gauge

A vibrating wire sensor is tested by steel string vibration. An experiment is conducted to characterize the force according to the variation in vibration frequency. In an actual output frequency signal, there is no strain gauge; it must be field calibration, signal drift, long distance transmission, and long time. The problem of poor durability was used, and the robustness was good [21]. This experiment solves the shortcoming of unstable strain gauges for a long time, and this product can be widely used in bridge monitoring.

A vibrating wire sensor has good measurement characteristics; it can achieve nonlinear characteristics of less than 0.1%, sensitivity of 0.05%, and less than 0.1%/10 °C temperature error.

After the strain gauge is manufactured, its steel string has a certain initial tensile force $T_{0}$ and thus has an initial frequency $F_{0}$. When the strain gauge is installed, the tensile force of vibrating wire changes with deformation, and the strain can be measured by the tensile force change of vibrating wire. Set the tension of the vibrating wire to T and the natural frequency to f. The relationship between tension and frequency can be expressed as Equation (1):

(1) $$T=Kf^{2}$$

where K is related to the length of string, and the mass per unit length can be expressed using Equation (2):

(2) $$\Delta T=T-T_{0}=K\left(f^{2}-f_{0}^{2}\right)$$

Assuming that the strain increment of strain gauge can be set to the strain increment of vibrating wire, Equation (3) can be derived as follows:

(3) $$\varepsilon_{h}=\varepsilon_{g}=\frac{\Delta K}{EA}$$

When EA is the axial stiffness of steel string, it can be derived from Equation (4) as follows:

(4) $$\varepsilon_{h}=\frac{K}{EA}\left(f^{2}-f_{0}^{2}\right)=k_{h}\left(f^{2}-f_{0}^{2}\right)$$

A mathematical model of the vibrating wire sensor can be expressed using Equations (5) and (6) as follows:

(5) $$F=K\left(f^{2}-f_{0}^{2}\right)$$

(6) $$F=A\left(f^{2}-f_{0}^{2}\right)+B\left(f-f_{0}\right)$$

**Figure 5:**
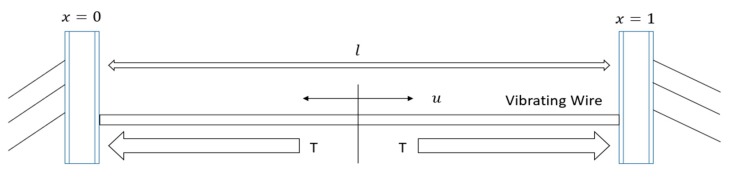
Working principle diagram of vibrating wire strain gauge.

When the length is such that the fine string of mass m is subjected to tension F (Figure 5), the natural frequency f can be expressed as Equations (7)–(9) as follows:

(7) $$f=\frac{1}{2}\sqrt{\frac{F}{ml}}$$

(8) $$f=\frac{1}{2l}\sqrt{\frac{ES\Delta l}{\rho l}}=\frac{1}{2l}\sqrt{\frac{E\Delta l}{\rho_{v}l}}$$

(9) f=φ(F)

Sensitivity can be derived using Equation (10) as follows:

(10) $$f^{2}=\frac{1}{4l^{2}}\frac{E\Delta l}{\rho_{v}l}=K\varepsilon$$

After differentiation, Equations (11) and (12) can be deduced as follows:

(11) 2fdf=Kdε

(12) $$k=\frac{df}{d\varepsilon }=\frac{K}{2f}$$

The material coefficient can be calculated using Equation (13):

(13) $$\{\begin{array}{c} K=\frac{1}{4l^{2}}\frac{E}{\rho_{v}}=\frac{ES}{4l^{2}\rho }\\ \varepsilon =\frac{\Delta l}{l} \end{array}$$

The above formula shows that sensitivity k is directly proportional to material coefficient K and inversely proportional to the vibration frequency of string.

After many experiments, the initial frequency is $f_{0}$ when the measured tension is $F_{0}$, and the vibration frequency is $f_{1}$ when the measured tension is $F_{1}=f_{0}+f$. The nonlinear error of vibrating wire strain gauge can be expressed using Equations (14) and (15) as follows:

(14) $$f_{1}=\frac{1}{2}\sqrt{\frac{F_{0}+\Delta F}{ml}}=\frac{1}{2}\sqrt{\frac{F_{0}}{ml}}\sqrt{1+\frac{\Delta F}{F_{0}}}=f_{0}\sqrt{1+\frac{\Delta F}{F_{0}}}=f_{0}\sqrt{1+\varepsilon_{F}}=f_{0}\;{\left(1+\varepsilon_{F}\right)}^{\frac{1}{2}}$$

(15) $$f_{1}=f_{0}\left(1+\frac{1}{2}\varepsilon_{F}-\frac{1}{8}\varepsilon_{F}^{2}+\frac{1}{16}\varepsilon_{F}^{3}-\cdots \right)$$

At that time $F_{2}=F_{0}-\Delta F$, leading to Equation (16):

(16) $$f_{2}=f_{0}\left(1+\frac{1}{2}\varepsilon_{F}-\frac{1}{8}\varepsilon_{F}^{2}+\frac{1}{16}\varepsilon_{F}^{3}-\cdots \right)$$

Its quadratic nonlinearity error can be expressed as Equation (17):

(17) $$\frac{\left|\frac{1}{8}f_{0}\varepsilon_{F}^{2}\right|}{\frac{1}{2}f_{0}\varepsilon_{F}}=\frac{1}{4}\varepsilon_{F}$$

The above formula shows that the larger $\varepsilon_{F}$, the larger $\delta_{m}$. At the same time, the ambient temperature mainly influences the frequency stability. The bulk density ρv and Δl caused by F do not change with the ambient temperature, so the frequency stability can be expressed using Equation (18):

(18) $$\gamma_{f}=\frac{df}{f}=\frac{dE}{2}E-\frac{3}{2}\frac{dl}{l}$$

When the ambient temperature changes, the vibrating wire strain gauge material and the measured structural material have different linear expansion coefficients, and the sensor is subjected to additional stretching or compression. The additional strain can be expressed as Equation (19):

(19) εT=(α−β)∙ΔT

where:

εT: Additional strain caused by temperature effect;
α: Linear expansion coefficient (°C^−1^) of the structural material to be tested;
B: The coefficient of linear expansion (°C^−1^) of a steel string of a vibrating wire strain gauge;
ΔT: Temperature change amount.

In actual application, the vibrating wire strain sensing system generally adopts software compensation. After the thermistor is set to collect the working environment temperature in the electromagnetic coil, the optimized temperature compensation algorithm is combined with the software to compensate in the demodulation instrument. The temperature strain compensation of the vibrating wire strain gauge mostly utilizes the two-dimensional regression method, polynomial fitting method, and neural network method. After the test results of the vibrating wire strain gauge are linearly fitted, the strain calculation method can be expressed as Equation (20):

(20) $$\varepsilon =a\times (f_{i}^{4}-f_{0}^{4})+b(f_{i}^{2}-f_{0}^{2})+k_{T}(T_{i}-T_{0})$$

where:

ε: The dependent variable of the current time relative to the initial position (10^−6^);
k: The steel string strain gauge minimum reading 10^−6^/(kHz^2^);
$f_{i}^{2}$: The Steel string strain gauge current output modulus kHz^2^;
$f_{0}^{2}$: The Steel string strain gauge initial output frequency modulus kHz^2^;
$k_{T}$: The steel string strain gauge temperature correction factor 10^−6^ °C;
$T_{i}$: The steel string strain gauge current time temperature value (°C);
$T_{0}$: Temperature value when measuring $f_{0}$ (°C).

When using polynomial fitting, the coefficients a and b were calculated using the least squares fitting method, and the strain calculation method for integrating temperature changes is shown in Equation (21):

(21) $$\varepsilon =a\times (f_{i}^{4}-f_{0}^{4})+b(f_{i}^{2}-f_{0}^{2})+k_{T}(T_{i}-T_{0})$$

The abovementioned various theoretical calculation methods and vibrating wire strain gauge measurement characteristics can better measure the strain, eliminate the strain generated by environmental influence, more accurately understand the mechanical strain of structure, and analyze the stress state of structural facility.

## 3. Test Plan

**Figure 6:**
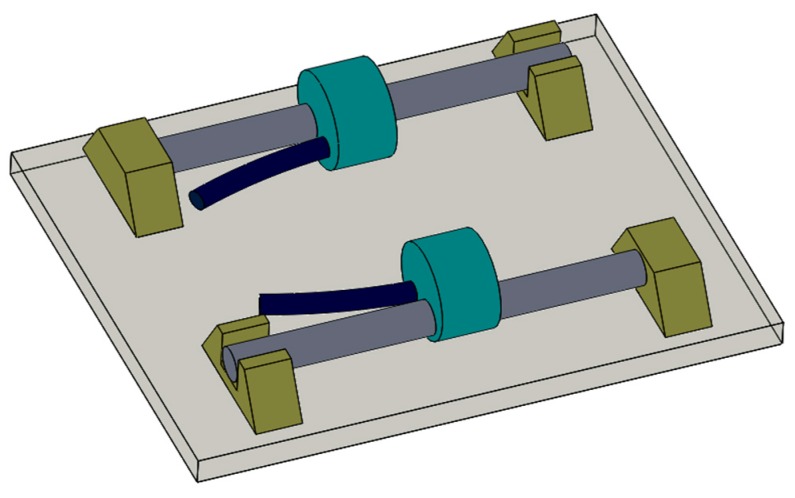
Caption for figure schematic diagram of the test fixture.

Two sensors were installed in parallel on the 45th steel tooling for free acquisition at the same frequency. The device (Figure 6) shows the material properties of No. 45 steel (Table 1). The acquisition device is equipped with a high-frequency dynamic acquisition device, which is a dynamic vibrating wire automatic acquisition system. This system uses a nonsweeping technology scheme to prevent the steel string vibration from attenuating, and an embedded mirror oscillation circuit to ensure excitation frequency. By matching the phase and true motion of steel cord and simultaneously detecting the resonant frequency of steel string through several waveform periods, noise immunity and resolution of measurement have largely improved compared with the flat-domain periodic averaging method.

The measurement module used the patented 8,671,758 products, and the dynamic measurement rate is 20 to 333 Hz. At the same time of dynamic measurement, the module also performs auxiliary measurement, performs static measurement at 1 Hz, provides finer measurement resolution, and better anti-interference to external noise source performance. The thermistor input signal of each vibrating string acquisition channel was measured at a high resolution of 24 bits at 1 Hz. The performance of thermistor parameters is shown in Table 2. The excitation module has a resolution of 26 mV and a dynamic measurement rate of 20, 50, 100, 200, and 333.33 bHz. The range of sensor resonance frequency is shown in Table 3. The measurement frequency accuracy is ± (0.005% reading + measurement resolution). The noise level corresponding to the measurement resolution b at different sampling rates is shown in Table 2, Table 3 and Table 4.

In the measurement, both steel plate and strain gauge will produce different deformations due to temperature changes. To minimize the acquisition error, a correlation curve between temperature and strain was analyzed. During the test, welding was used. To solder the two sensor holders on the tooling and for better verification, the effect of temperature response, and correlation, when collecting the zero point, one sensor was set to the free state, one sensor passed the fixture. The force was applied such that the sensor zero point acquisition and the first sensor’s zero point acquisition strain difference is about 140 με.

## 4. Analysis of Test Data

The relationship between temperature and strain measurement shows that the strain changes correspondingly when the temperature changes. The strain reversely sways as the temperature changes. We got a good corresponding relationship. The temperature and strain data collected using the two sensors A and B were compared, and the correlation between the two data was analyzed. During the analysis, there are usually two ways of correlation: Autocorrelation and cross-correlation. The autocorrelation function is known as the autocorrelation equation and used to describe the correlation between the correlation functions of related data at different times as shown in Equation (22):

(22) $$R_{f}(\tau )=f(\tau )*f^{*}\left(-\tau \right)=\int_{-\infty }^{\infty }f(t+\tau )f^{*}\left(t\right)dt=\int_{-\infty }^{\infty }f(t)f^{*}\left(t-\tau \right)dt$$

At the same time, cross-correlation or cross-covariance were also used to represent a measure of similarity between two signals. Cross-correlation mainly analyzes the degree of correlation between two time series. Cross-correlation is essentially similar to the convolution of two functions. For discrete functions $f_{i}$ and $g_{i}$, it can be defined as Equation (23):

(23) $${\left(f*g\right)}_{i}\equiv \sum_{j}f_{j}^{*}g_{i+j}$$

If the continuous signal is set to two sets of f(x) and g(x), then the cross-correlation is defined as Equation (24):

(24) $$(f*g)\left(x\right)\equiv \int f^{*}\left(t\right)g\left(x+t\right)dt$$

**Figure 7:**
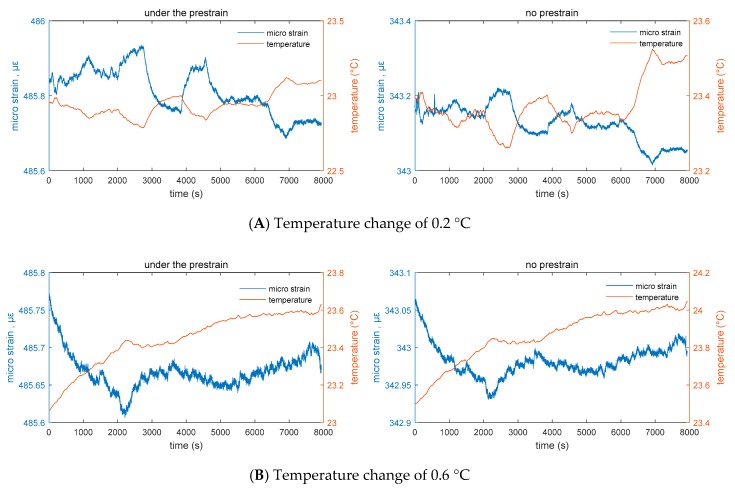

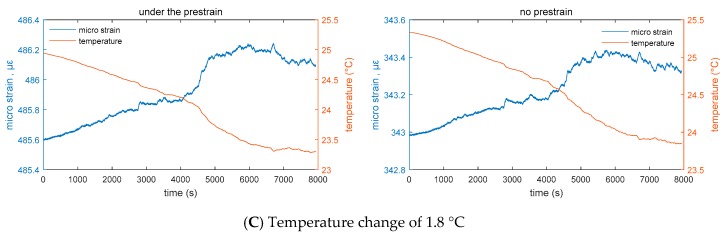
Free state strain values of strain sensors A and B in a temperature change of 0.2, 0.6, and 1.8 °C.

During this test analysis, the data collected using the two sensors A and B were compared, and the correlation between the two sets of data was analyzed. Their similarities were derived to verify the accuracy and reliability of dynamic acquisition of data obtained using sensors A and B. The correlation coefficient between the temperature data of sensors A and B was calculated to be 0.9983, and the correlation degree is highly correlated. The correlation coefficient between the strain data of sensors A and B is 0.9895, and the correlation degree is highly correlated. Cross-calculation can be obtained. The correlation between temperature and strain of sensor A is −0.6683, and the correlation between temperature and strain of sensor B is −0.5573. The experimental results show that the values are significantly correlated (Figure 7).

During the test, to better use the knowledge of metrology to analyze the dynamic RMS change output, verify the reliability of linear relationship between temperature and strain monitoring, and repeat the calculation of temperature measurement and strain measurement, the experiment measured the comprehensive reflection of various random influencing factors, including the instability of tooling materials used, random error caused by the laying process, instability of sensing instrument, environmental conditions, and other factors, as well as the actual measured randomness. The measured object also affects the dispersion of measured values, especially when the random variation in the measured object during the bridge monitoring process is large. Therefore, the dispersion of experimentally measured values is typically slightly greater than the dispersion introduced by the sensor static calibration standard itself. To be less affected by outliers, all the deformations and the corresponding temperature were measured. During the calculation of repeatability of the experiment, the arithmetic mean value of measured value should be calculated first, and the calculation formula can be expressed as Equation (25):

(25) $$\bar{F}=\frac{\sum_{i=1}^{n}F_{i}}{n}$$

where:

$F_{i}$—The strain measurement indication of the *i*-th measurement, *με*;
n—The number of measurements.

The measurement repeatability can be quantitatively expressed using the experimental standard deviation Sr(y), and the calculation formula can be expressed as Equation (26):

(26) $$\mathrm{Sr}\;(y)=\sqrt{\frac{\sum_{i=1}^{n}{\left(F_{i}-\bar{F}\right)}^{2}}{n-1}}$$

where:

$F_{i}$—The measured strain measured at the *i*-th measurement, *με*;
$\bar{F}$—The arithmetic mean of indications of strain measurement, *με*;
n—number of measurements.

Using the above formula, under the synchronous acquisition test conditions, sensors A and B were repetitively used for the temperature and strain measurements, respectively. The repeatability of sensor A strain indication is 0.16071, and the temperature measurement repeatability is 0.09971. The repeatability of strain measurement of sensor B is 0.11743, and the repeatability of temperature measurement is 0.08209. The repeatability shows that the stability of sensor B is better, and the experiment can be performed under the condition that the initial state of sensor is small. The measured values are more stable and more clearly characterized by the corresponding relationship between temperature and strain.

**Figure 8:**
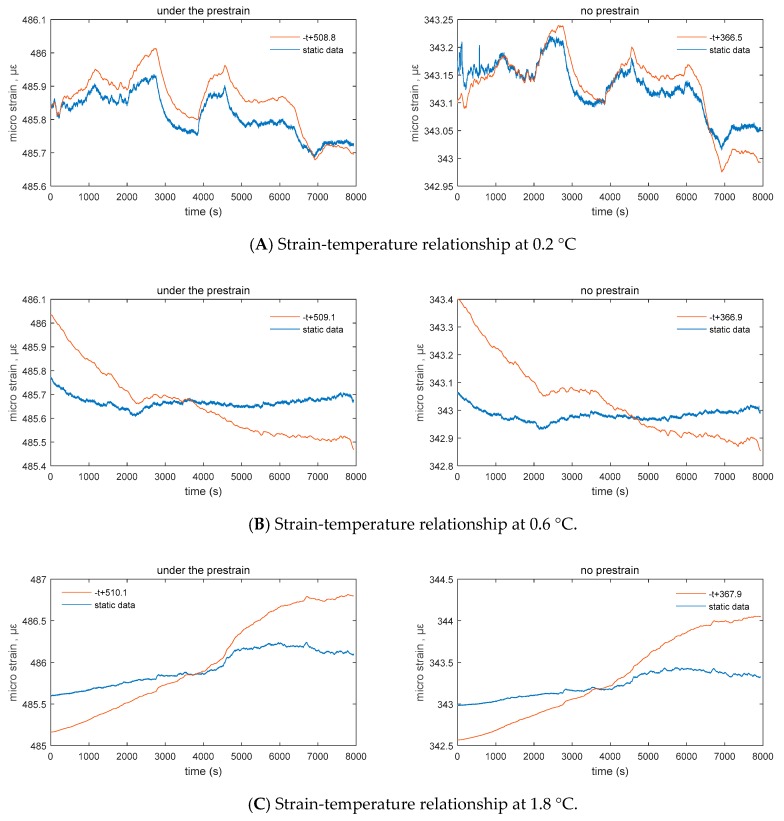
Strain-temperature relationship between 0.2, 0.6, and 1.8 °C intervals for strain gauges A and B.

At the same time, the relationship between strain and temperature was analyzed during a temperature variation of 0.2, 0.6, and 1.8 °C. Figure 4, Figure 5 and Figure 6 show that the correlation did not change with the change in temperature. A stable negative correlation curve relationship was maintained. Among them, sensor A has a relatively weak induction of temperature and deformation under the prestrain of 140 με, which can be characterized as shown in the figure. In the absence of external excitation, the change in experimental numerical temperature is less than the perception of sensor B. At the same time, the corresponding strain produces a small change in the output. Sensor A temperature and the strain relationship map shows that in the free state of acquisition device, the experimental temperature will also have a corresponding change during small changes. However, both sensors A and B can consistently respond to the corresponding output temperature and strain signals (Figure 8).

After calculating different temperature changes, the experimental values show the correlation between strain and temperature. By analyzing the strain-temperature correlation function, it can be found that the temperature change is less than that of sensor B when there is no external excitation. At the same time, the change in output corresponding to the strain is small. In different temperature variation ranges, the experimental data were used to analyze the relationship between the temperature and strain of sensor A. It was observed that the acquisition device is in a free state, and the temperature is small. The effect of corresponding change will be produced. According to statistical analysis, the strain-temperature correlation can be expressed as Δμε=ΔT+510, and the strain measurement result has a negative correlation with the temperature change. From the relationship between the temperature and corresponding strain curves of sensor B in the free state, it can be observed that the strain-temperature correlation can be expressed as Δμε=ΔT in the state where sensor B is more free under the same conditions as sensor A. Δμε=ΔT+367; the strain measurement results have a stable negative correlation with temperature change. During data acquisition, it was found that strain gauge A is larger at the same temperature due to prestressing, the strain output is larger than the output of sensor B, and the correlation between temperature, peak, and valley is more prominent. It was assumed that 0.2 is a one-fold change in temperature. During the three-fold change and nine-fold change, the steady-state strain curve is stable, and the correlation function is not fluctuating. However, in the state where the prestrain of sensor A is 140 με, the induction of temperature and deformation is relatively weak, and the difference is about 3–6 με, which is about 2–5% of the prestress, as shown in Figure 7 and Figure 8. In the later test, more theoretical and experimental verifications are needed. Combined with the artificial intelligence model training method, the expansion effect caused by the material itself after the vibration of vibrating string was analyzed, and the environmental impact error was maximized. The tooling surface was optimized for temperature conductivity. Since this test is the calibration test for the maximum fitting, instrumentation error, etc., the welding process was used, so that the above effects can be neglected. At the same time, referring to Table 4, the noise of the device is 9.775 × 10^−9^ με, which can be ignored. Through comprehensive evaluation, it was found that the method of synchronously collecting sensors at similar positions can achieve the online comparison of the corresponding variable measuring sensors. The later test can be calibrated by high-frequency dynamic acquisition to calibrate the low-frequency acquisition and combined with the relevant optimization algorithm for calculation. The maximum optimization restores the original deformation magnitude, and at the same time reduces the instability of constitutive performance parameters such as the stiffness and strength of structure. The experimental results show the calibration of the corresponding variable monitoring sensor.

## 5. Conclusions

In engineering, the strain test method is usually used to determine the actual stress state of structure and monitor the structural deformation increment, and the structural strain test is an important method to solve the structural strength problem. During strain detection and monitoring, the calibration of strain gauge plays a significant role in the accuracy of data. To ensure the accuracy of traceability of magnitude, we should study the corresponding relationship between the temperature and strain of strain gauge and the online calibration test method, which is important for the online metrological traceability system of strain monitoring sensor. We focused on researching the influence of temperature and prestress effect on sensors by analyzing differences of their measurement results in specified situation. In this study, a dynamic vibrating wire measurement module was used to dynamically collect the temperature and strain of two vibrating wire sensors in the free state, and the correlation between temperature and strain was analyzed. The correlation coefficient between the temperature data of sensors A and B were statistically analyzed. The degree of relevance 0.9983 is highly correlated. The experimental results show that the correlation coefficient between the strain data of sensors A and B is 0.9895, and the correlation degree is highly correlated. After cross-calculation, a significant correlation was observed between temperature and strain indications. An experiment was carried out to analyze the measurement repeatability by using the knowledge of metrology. After analysis, it was found that the repeatability of temperature and strain values is within 0.2, and the consistency between the measured results of the same measurement is good. At the same time, the relationship between strain and temperature in different temperature variation intervals was analyzed. It was found that the temperature change has a small effect on the strain in the prestressed state, but the relationship between temperature and strain is relatively stable. Finally, the experiment verified the feasibility of parallel measurement scheme corresponding to the online calibration of variable monitoring sensor and analyzed the effect of temperature corresponding to the variable measurement under different prestress conditions, providing theoretical and experimental verification support for establishing an online metrological verification standard. This study can also better confirm the accuracy of bridge health monitoring data, effectively reduce the monitoring error caused by temperature drift, effectively eliminate the impact of environmental load, and better feedback the essential deformation of bridge structure. Considering the reliability risk of sensors after a long-time work period, such as signal drift, the approach proposed in this paper can be used as a substitute to such strain monitoring system in order to determine whether these sensors should be replaced. We expect our approach to be expandable to various applications. It can provide important support for the effective monitoring data extraction of the bridge health monitoring system.

## Figures and Tables

**Table 1:** Forty-five steel material characteristics.

Content	Value and Unit
Density	7.85 g/cm^3^
Modulus of elasticity	210 GPa
Poisson ratio	0.269
Tensile strength	600 MPa
Yield strength	355 MPa
Elongation	16%
Section shrinkage	40%
Impact work	39 J

**Table 2:** Thermistor performance parameters.

Content	Value and Unit
Half bridge arm	0.1% accurate resistance is 4.99 KΩ
Excitation voltage	1.5 V
Resolution	0.002 Ω RMS @ 5 KΩ thermistor
Accuracy (−55–85 °C)	0.15% of reading
Measurement rate	1 Hz

**Table 3:** Range of sensor resonance frequencies.

Sample Rate (Hz)	Minimum Sensor Frequency (Hz)	Maximum Sensor Frequency (Hz)
20	290	6000
50	290	6000
100	580	6000
200b	1150	6000
333b	2300	6000

**Table 4:** Measurement resolution b (typical value for a 2.5 kHz resonant frequency sensor).

Sample Rate (Hz)	Noise Level (Hz RMS)
1	0.005
20	0.008
50	0.015
100	0.035
200C	0.11
333C	0.45

## References

[1] Mao J.X., Wang H., Feng D.M., Tao T.Y., Zheng W.Z. (2018). Investigation of Dynamic Properties of Long-Span Cable-Stayed Bridges Based on One-Year Monitoring Data Under Normal Operating Condition. Struct. Control Health Monit..

[2] Wang H., Zhang Y.M., Mao J.X., Wan H.P., Tao T.Y., Zhu Q.X. (2019). Modeling and Forecasting of Temperature-induced Strain of a Long-span Bridge using an Improved Bayesian Dynamic Linear Model. Eng. Struct..

[3] Mao J.X., Wang H., Li J. (2019). Fatigue Reliability Assessment of a Long-Span Cable-Stayed Bridge Based on One-Year Monitoring Strain Data. J. Bridge Eng..

[4] He H., Wang W., Tian D., Sun J., Xiong C. (2010). Optimization of Vibrating Wire Sensor Excitation Strategy. Chin. J. Sens. Actuators.

[5] Wen Z., Xia Z., Li F. (2012). Research on Frequency Measurement Technology of String Vibration Sensor. J. Xi’an Poly. Univ..

[6] Tian Z., Huang Z. (2016). Nonlinear Compensation of Vibrating Wire Sensor Based on Spline Interpolation and Least-Squares Method. Meas. Control Technol..

[7] Chen N., Li H., He H., Xie K. (2017). An Self-Adaptive Vibrating Pick-up Method for Vibrating Wire Sensor. J. Guangxi Univ. (Nat. Sci. Ed.).

[8] Xu Y., Yang X., Wei T., Yao J. (2018). Analysis of Strain Transfer Influence Factors of Resistance Strain Sensor. China Meas. Test.

[9] Zhang Y., Liang P., Zhang Y., Chen W., Huang M. (2011). Design of Calibration Device for Vibrating Wire strain Transducer. Ind. Instrum. Autom..

[10] Guangzhou Guangcai Testing Instrument Co., Ltd (2016). Vibrating Wire Strain Gauge Calibration Instrument: China.

[11] Bai S., Xiao Y., Huang B., Liu G. (2016). Research on Strain Calibration Method of Fiber Bragg Grating Sensor. J. Vib. Mea. Diagn..

[12] Zhang H., Nie F., Fan D. (2017). Calibration Method, Device and System of Fiber Bragg Grating Sensor.

[13] Chen C., Yan D., Chen Z., Tu G., Tian Z. (2004). Technique Research of Vibrational Chord Strain Gauge to Concrete. China J. Highw. Transp..

[14] Bai T., Deng T., Xie J., Hu F.P. (2005). Accurate Mathematical Model of Vibrating Wire Sensor and Its Application. Chin. J. Rock Mech. Eng..

[15] Iadicicco A., Campopiano S. (2015). Sensing Features of Long Period Gratings in Hollow Core Fibers. Sensors.

[16] Wang Y.B., Zhao R.D., Chen L., Xu Y., Xie H.Q. (2017). Temperature Correction Test of Vibrating Wire Strain Sensor. J. Archit. Civ. Eng..

[17] Lee H.M., Park H.S. (2013). Measurement of Maximum Strain of Steel Beam Structures Based on Average; Strains from Vibrating Wire Strain Gages. Exp. Technol..

[18] Choi S.W., Kwon E., Kim Y., Hong K., Park H.S. (2013). A Practical Data Recovery Technique for Long-Term Strain Monitoring of Mega Columns during Construction. Sensors.

[19] Park H., Lee H., Choi S., Kim Y. (2013). A Practical Monitoring System for the Structural Safety of Mega-Trusses Using Wireless Vibrating Wire Strain Gauges. Sensors.

[20] Barot D., Wang G., Duan L. (2019). High-Resolution Dynamic Strain Sensor Using a Polarization-Maintaining Fiber Bragg Grating. IEEE Photonics Technol. Lett..

[21] Jin X., Sun C., Duan S., Liu W., Li G., Zhang S., Chen X., Zhao L., Lu C., Yang X. (2019). High Strain Sensitivity Temperature Sensor Based on a Secondary Modulated Tapered Long Period Fiber Grating. IEEE Photonics J..

